# Seven-Valent Pneumococcal Conjugate Vaccine and Nasopharyngeal Microbiota in Healthy Children

**DOI:** 10.3201/eid2002.131220

**Published:** 2014-02

**Authors:** Giske Biesbroek, Xinhui Wang, Bart J.F. Keijser, Rene M.J. Eijkemans, Krzysztof Trzciński, Nynke Y. Rots, Reinier H. Veenhoven, Elisabeth A.M. Sanders, Debby Bogaert

**Affiliations:** University Medical Center Utrecht, Utrecht, the Netherlands (G. Biesbroek, X. Wang, K. Trzciński, E.A.M. Sanders. D. Bogaert);; TNO, Zeist, the Netherlands (G. Biesbroek, B.J.F. Keijser);; Julius Center for Health Sciences and Primary Care, Utrecht (R.M.J. Eijkemans);; Netherlands Vaccine Institute, Bilthoven, the Netherlands (N.Y. Rots);; Spaarne Hospital, Hoofddorp, the Netherlands (R.H. Veenhoven)

**Keywords:** seven-valent pneumococcal conjugate vaccine, PCV-7, pneumococcal conjugate vaccine, pneumococcal conjugate vaccination, pneumococci, bacteria, respiratory tract, colonization, randomized controlled trial, nasopharyngeal microbiota, children

## Abstract

Seven-valent pneumococcal conjugate vaccine (PCV-7) is effective against vaccine serotype disease and carriage. Nevertheless, shifts in colonization and disease toward nonvaccine serotypes and other potential pathogens have been described. To understand the extent of these shifts, we analyzed nasopharyngeal microbial profiles of 97 PCV-7–vaccinated infants and 103 control infants participating in a randomized controlled trial in the Netherlands. PCV-7 immunization resulted in a temporary shift in microbial community composition and increased bacterial diversity. Immunization also resulted in decreased presence of the pneumococcal vaccine serotype and an increase in the relative abundance and presence of nonpneumococcal streptococci and anaerobic bacteria. Furthermore, the abundance of *Haemophilus* and *Staphylococcus* bacteria in vaccinees was increased over that in controls. This study illustrates the much broader effect of vaccination with PCV-7 on the microbial community than currently assumed, and highlights the need for careful monitoring when implementing vaccines directed against common colonizers.

## Abstract 2

Careful monitoring of vaccines against common bacterial colonizers is needed.

Vaccination is one of the most effective methods to prevent infectious diseases by direct protection of persons against a specific pathogen and by eradication of these specific pathogens from the population, leading to so called herd effects or indirect protection (*1*). Over the past decade, a 7-valent pneumococcal conjugate vaccine (PCV-7) was introduced in national immunization programs for newborns in most high-income countries, and the newer-generation 10-valent and 13-valent vaccines are being progressively introduced in developing countries (*2*).

The specific serotypes of the first licensed 7-valent pneumococcal vaccine are common colonizers of the upper respiratory tract of children during the first years of life, in which these serotypes generally reside as part of the nasopharyngeal microbiota (bacterial community) (*3*). However, these bacteria might occasionally spread beyond this niche and cause otitis media, pneumonia, sepsis, or meningitis (*4*). Vaccines show effectiveness against vaccine-serotype disease, nasopharyngeal acquisition of pneumococci, and pneumococcal transmission. However, nonvaccine pneumoccal serotypes fill the vacant nasopharyngeal niche, leaving overall pneumococcal carriage similar or only temporarily decreased (*5*,*6*) and lead to a gradual increase in nonvaccine serotype disease (*7*). In addition, several studies have raised awareness of the replacement of vaccine serotypes in the bacterial community with other potential pathogens, such as *Haemophilus influenzae* and *Staphylococcus aureus* in carriage or disease (*8*–*11*). This replacement is likely explained by the highly interactive nature of the microbiota in the natural habitat of the specific bacterium (*12*).

 The recent availability of high-throughput, deep-sequencing techniques has made it possible to obtain more insight in the microbiota in humans, including the not yet cultivated fraction of bacteria. These techniques have elucidated that bacteria of the human microbiota outnumber human host cells by 10-fold, and microbiota composition varies greatly between body sites and persons. Colonization is a dynamic process of interactions among microbes and between microbes and the host and result in balanced bacterial ecosystems that benefit health. Perturbations of these interactive microbial structures (e.g., by environmental change or vaccinations) alter the bacterial network structures and may thereby influence the presence and containment of other microbiota members, and these alterations have effects on health and susceptibility to disease (*13*,*14*).

Given the changes in pneumococcal serotypes, as well as well as *S. aureus* and *H. influenzae* carriage after vaccination with PCV-7 (*7*,*8*), we questioned whether the effects of PCV-7 could be even more extensive than initially believed. We therefore studied the effects of PCV-7 on the complete nasopharyngeal microbiota of healthy children in a randomized controlled trial by using deep-sequencing techniques. The study was initiated shortly before nationwide implementation of PCV-7 in the Netherlands, therefore before herd effects appeared, which enabled us to measure the direct effects of the vaccine (*15*).

## Methods

### Study Design and Population

Nasopharyngeal samples were obtained from a randomized controlled trial that studied efficacy of reduced-dose schedules of PCV-7 on pneumococcal carriage in 1,005 healthy children in the Netherlands. The methods of this trial have been described (*15*). In brief, participants were randomly assigned to receive 1) PCV-7 at 2 and 4 months of age (2-dose group) of age; 2) PCV-7 at 2, 4, and 11 months of age (2 + 1-dose group); or 3) no PCV-7 (unvaccinated control group).

For the present study, we selected nasopharyngeal samples of the group of children that received 3 vaccinations with PCV-7 (n = 336) and of the group of children that received no vaccinations with PCV-7 (controls) (n = 331). To avoid seasonal influences on microbiota composition (*3*), we selected samples from children whose first birthday was during October 2006–January 2007. To avoid interference from background DNA, only samples from those children with sufficient bacterial density at 12 and 24 months of age (i.e., samples with DNA levels ≥1 pg/µL) were selected for 454 pyrosequencing, as described (*16*).

Nasopharyngeal swab specimens from the controlled trial had been obtained during home visits by using a deep transnasal approach with a flexible, sterile, dry, cotton-wool swab (TranswabPernasal Plain; Medical Wire and Equipment Co., Ltd., Corsham, UK). Specimens were immediately inoculated into transswab modified Amies Medium, 483CE (Copan Diagnostics Inc., Murietta, CA, USA), transported to the laboratory, and stored in saline within 24 h at −80°C until further analyses. All nasopharyngeal swab specimens were cultured for *H. influenzae, Moraxella catarrhalis, S. aureus*, and *Streptococcus pneumoniae* and subjected to pneumococcal serotyping (*15*,*17*,*18*). With each nasopharyngeal swab specimen, a questionnaire on risk factors for pneumococcal carriage in children and prior antimicrobial drug use was completed.

The randomized controlled trial (NCT00189020) was approved by an acknowledged Dutch National Ethics Committee (Stichting Therapeutische Evaluatie Geneesmiddelen) and conducted in accordance with European Statements for Good Clinical Practice, which included the provisions of the Declaration of Helsinki of 1989. Before enrollment, written informed consent was obtained from both parents of each participant.

### Construction of Phylogenetic Library

The selected subset was processed for sequencing of the 16S rDNA gene; the 454 GS-FLX-Titanium Sequencer (Life Sciences, Branford, CT, USA) was used for sequencing. The 16SrDNA gene is a conserved gene with variable regions among bacteria. Therefore, sequencing of this gene enables detection of all bacteria in the microbiota, which enables discrimination between bacterial taxa. DNA was extracted and quantified by quantitative PCR specific for conserved regions of the 16S rDNA gene. A barcoded amplicon library was generated by amplification of the V5–V7 hypervariable region of this gene and sequenced unidirectional, which generated ≈1.5 million sequences. Details of the methods have been described (*3*,*16*) and are shown in the Technical Appendix. The obtained sequences were processed and classified by using modules implemented in the Mothur V.1.20.0 software platform (*19*–*22*). This platform enables sequence classification on several taxonomic levels on the basis of evolutionary relatedness. The smallest accurate taxonomic level obtained by using 16S rDNA gene sequencing is the operational taxonomic unit (OTU), which is based on 97% similarity in nucleotide composition and enables differentiation just beyond genus level: OTUs do not always discriminate between species, and multiple OTUs might represent a specific genus, each capturing distinct lineages within it.

For each of the samples, rarefaction curves were plotted and sequence coverage was calculated by using the formula 1 – (number of OTUs with a single sequence per sample/number of samples in the study) to ensure that sufficient sequence numbers were analyzed. Sequence data were subjected to weighted UniFrac analysis by using the UniFrac module implemented in Mothur (*23*). The UniFrac metric is a proxy for the distance between microbial communities based on evolutionary relatedness of lineages in each sample. For all samples, we calculated the presence and relative and absolute abundance of all OTUs. The relative abundance was calculated as the proportion of sequences assigned to a specific OTU divided by the overall number of obtained sequences per sample. In addition, for the absolute abundance, we multiplied the relative abundance of an OTU by the obtained bacterial load per sample measured by quantitative PCR.

### Statistical Analyses

Data analyses were performed by using R version 2.7 (http://cran.r-project.org/bin/windows/base/old/2.7.1/), Excel 2011 (Microsoft, Redmond, WA, USA), and SPSS version 15.0 (SPSS Inc., Armonk, NY, USA). We used the Pearson χ^2^ test to compare baseline characteristics between PCV-7–vaccinated children and control children. To visualize the weighted UniFrac dendrogram in relation to metadata, we used iTOL version 2 software (*24*). We used univariate and multivariate linear regression models (function Im and analysis of variance in software package R) to study the effect of vaccination with PCV-7 on microbiota profiles. We adjusted for antimicrobial drug use 1 month before sampling, the presence of siblings, and daycare attendance in all multivariate linear regression models. Associations were considered statistically significant after correction for multiple testing by determining the false-discovery rate (q value 0.2). Relative effect sizes and their 95% CIs were calculated for all significant OTUs from the standardized regression coefficients of the fitted linear model, whereby 1 indicates no effect, >1 indicates higher abundance, and <1 indicates less abundance in vaccinated children than in controls.

Interindividual variability between vaccinated and control children at 12 months and 24 months of age was calculated by using Pearson correlations and tested for significance by using the Mann-Whitney U test. We used nonmetric multidimensional scaling (nMDS) to compare microbiota profiles for dissimilarities and Euclidean distances to locate each sample in a low-dimensional space. OTUs were clustered hierarchically by using average linkage and Pearson correlation. The optimal number of clusters was identified by using the Silhouette index. OTU clusters and Pearson correlations between OTUs were displayed by using Cytoscape V2.8.2 (*25*).

## Results

### Characteristics of Study Population and Culture Results

We sequenced the nasopharyngeal microbiota of 97 children at 12 and 24 months of age who had received PCV-7 at 2, 4, and 11 months of age, and 103 controls. Similar to the main trial (*15*), in this subset of children, baseline characteristics were not different between PCV-7 vaccinees and controls (Table). Also, use of antimicrobial drugs was low, especially in the month before sampling, and no correlation was observed between antimicrobial drug use and vaccination with PCV-7 (partial correlation, r<–0.01).

**Table:** Baseline characteristics of PCV-7 vaccinated and unvaccinated children (controls) at 12 and 24 months of age*

Characteristic	12 mo				24 mo		
	PCV-7, n = 97	Control, n = 103	p value		PCV-7, n = 97	Control, n = 103	p value
Male sex	54 (56)	53 (51)	NS		54 (56)	53 (51)	NS
Crowding							
Presence of siblings in household	52 (54)	57 (55)	NS		64 (66)	65 (63)	NS
Daycare attendance†	62 (64)	68 (66)	NS		75 (77)	74 (72)	NS
Symptoms of URTI							
URTI (<6 mo)	56 (58)	55 (54)	NS		41 (42)	43 (42)	NS
Current cold‡	40 (41)	38 (37)	NS		36 (37)	39 (38)	NS
Current otitis media	3 (3)	6 (6)	NS		0	0	NS
History of wheezing	15 (16)	13 (13)	NS		10 (10)	9 (9)	NS
Smoke exposure at home	6 (6)	7 (7)	NS		6 (6)	6 (6)	NS
Antimicrobial drug use (<1 mo)§	4 (4)	5 (5)	NS		2 (2)	2 (2)	NS
Carriage detected by culture							
* Streptococcus pneumoniae*	63 (64.9)	80 (77.7)	0.06		59 (60.8)	75 (72.8)	0.10
Vaccine serotypes	30 (30.9)	51 (49.5)	0.01		16 (16.5)	42 (40.8)	0.001
Nonvaccine serotypes	33 (34.0)	29 (28.2)	NS		43 (44.3)	33 (32.0)	0.08
* Moraxella catarrhalis*	75 (77.3)	85 (82.5)	NS		67 (96.1)	81 (78.6)	NS
* Haemophilus influenzae*	56 (57.7)	50 (48.5)	NS		55 (53.4)	59 (57.3)	NS
* Staphylococcus aureus*	9 (9.3)	5 (4.9)	NS		7 (6.8)	4 (3.9)	NS

*Values are no. (%) unless otherwise indicated. p values were calculated by using the χ^2^ test and are shown when there was a trend (p = 0.05–0.1) or a significant difference (p<0.05). PCV-7, 7-valent pneumococcal conjugate vaccine; NS, not significant (p>0.1); URTI, upper respiratory tract infection. †Defined as at least 4 h/wk of daycare with >1 child from a different family. ‡Presence of mild symptoms of a respiratory tract infection at time of sampling as reported by parents. §Defined as use of antimicrobial drug within 1 mo before sampling.

Consistent with the main trial, vaccine serotype pneumococcal carriage decreased in PCV-7–vaccinated children. However, because of a lower number of children than in main trial and loss of statistical power, we observed only a trend toward increased carriage of nonvaccine-type pneumococci (p = 0.08) at 24 months of age. Furthermore, the increase in *S. aureus* carriage at 12 months of age in vaccinees observed in the main trial was not significant in this subset because of a loss of power (Table) (*18*,*26*).

### Sequence and Microbiota Characteristics

**Figure 1:**
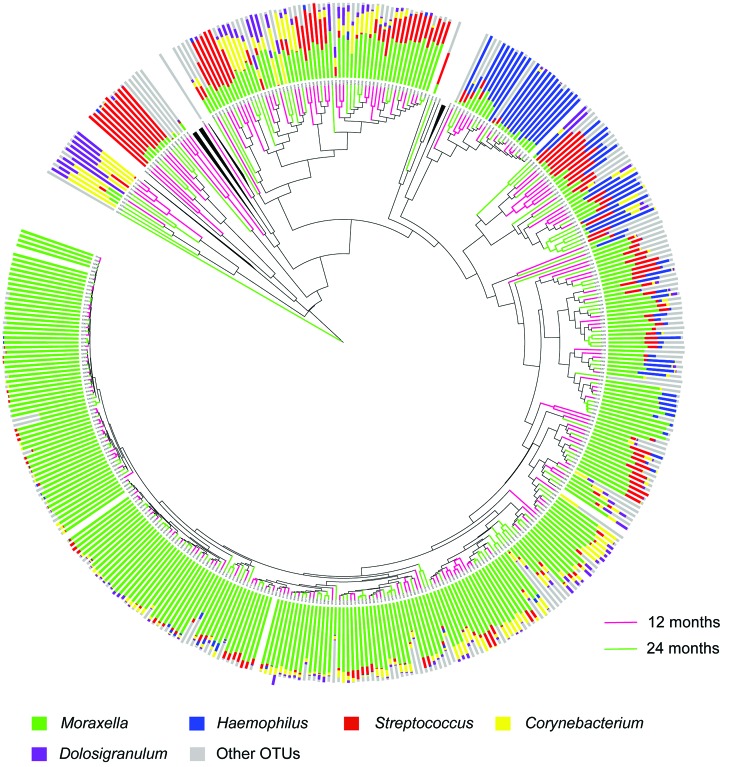
Weighted UniFrac analyses (*23*) of nasopharyngeal samples of children at 12 and 24 months of age vaccinated with 7-valent pneumococcal conjugate vaccine. Clustering of samples was based on evolutionary (phylogenetic) relatedness by using Weighted UniFrac analyses. Clustering is shown in a circle dendrogram. Each branch represents a sample and each adjacent histogram represents the relative abundance of the top 5 operational taxonomic units (OTUs) found in that sample. Differences in length of branches among samples reflect their distance (i.e., dissimilarity) to each other. Branches of reference samples were collapsed and are represented by black triangles. Samples are mostly dominated by *Moraxella, Streptococcus*, and *Haemophilus* spp., or the combination of *Dolosigranulum* and *Corynebacterium* spp., which highly affects sample clustering by Weighted UniFrac. Branches are colored according to age of sampled children (purple = 12 months, green = 24 months). No clear clustering of samples by age was observed.

We obtained 1,016,934 high-quality sequences (mean ± SD 2,561 ± 767 sequences/sample). Sequence depth was sufficient to obtain a high degree of sequence coverage for all samples (mean 0.995, median 0.996, range 0.975–1). Sequencing of nasopharyngeal microbiota identified a diverse ecosystem dominated by the well-known bacterial genera *Moraxella*, *Streptococcus*, and *Haemophilus*, but also *Dolosigranulum* and *Corynebacterium* (Technical Appendix Table 1). In addition, we detected a range of lower abundant bacterial genera (424 OTUs excluding singletons), present in either many (*Escherichia*/*Shigella*, *Neisseria*, and *Gemella* spp.) or few (*Sneathia* and *Porphyromonas* spp.) children. We found that for most children, the microbiota profile was determined mostly by abundance of the 5 predominant OTUs, and did not differ in children at 12 and 24 months of age (Figure 1).

To discriminate potential pathogens in the OTU set, we correlated the culture results of the samples with the corresponding OTUs of *Moraxella*, *Streptococcus*, *Haemophilus*, and *Staphylococcus*. We observed a strong correlation between culture results and the highest ranking OTUs for the respective genera (p<0.005) (Technical Appendix Table 2), which indicated a strong representation of these potential pathogens within these OTUs.

### Nasopharyngeal Microbiota Composition in Vaccinees and Controls

**Figure 2:**
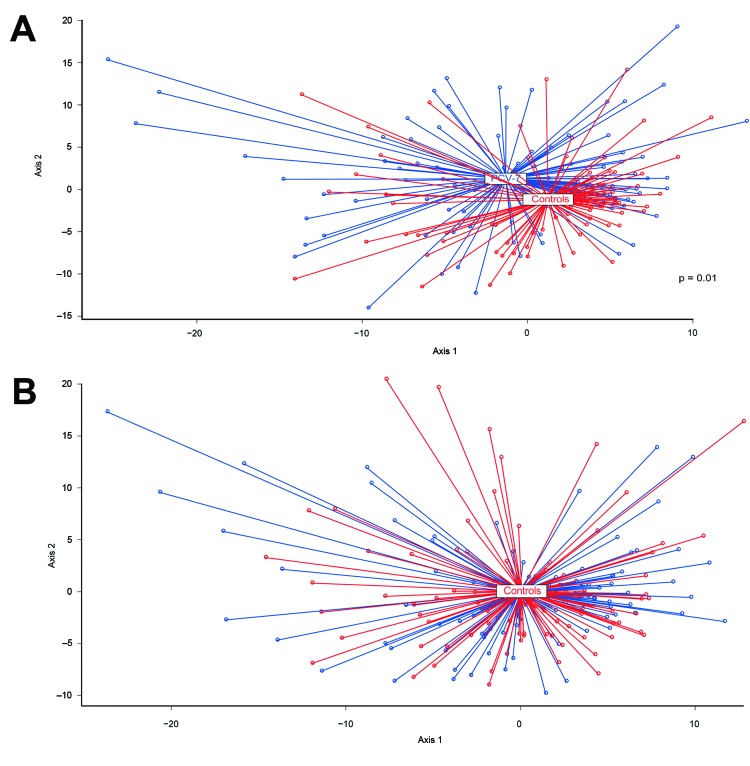
Nonmetric multidimensional scaling (nMDS) of microbiota profiles of children vaccinated with 7-valent pneumococcal conjugate vaccine and control children at 12 and 24 months of age. Microbiota profiles were compared between groups by using nMDS to find dissimilarities between samples and locate samples in a 2-dimensional space. Each circle represents the microbiota profile of a sample. Boxes indicate geometric means of both groups in which the length of the line between the sample (circle) and the geometric mean (box) indicates the distance of that sample from the geometric mean. Longer lines indicate higher distances of samples (i.e., higher variability between sample compositions). A) nMDS plots of vaccinated children (blue lines) and controls (red lines) at 12 months of age. The geometric mean of microbiota profiles differed significantly (p = 0.01, by F-test) between vaccinated children and controls. B) nMDS plots of vaccinated children (blue lines) and controls (red lines) at 24 months of age, showing no differences in geometric means of microbiota profiles between the 2 groups.

To evaluate the effect of vaccination with PCV-7 on the overall microbial community composition, we first calculated the degree of dissimilarity in microbiota composition between vaccinees and controls by using nMDS (*27*). We observed a significant shift in microbiota profiles between vaccinated and nonvaccinated children at 12 months of age (geometric means; p = 0.01, by F-test) but not at 24 months of age (Figure 2).

Because nMDS suggested higher variability of community profiles in vaccinees, we calculated interindividual variability in microbiota composition among vaccinees and controls by using Pearson correlations. We confirmed higher interindividual variability (i.e., less similarities between profiles) among vaccinees than in control children at 12 months of age (median correlation coefficient r = 0.39 vs. 0.41, p<0.0001) than at 24 months of age (median correlation coefficient r = 0.42 vs. 0.44, p = 0.006). At 12 months of age, this variability was accompanied by a significantly higher number of OTUs per community profile (i.e., higher diversity of bacteria) in vaccinated children (median 20, range 6–82) than in unvaccinated controls (median 17, range 4–46; p = 0.002) (Technical Appendix Table 3).

**Figure 3:**
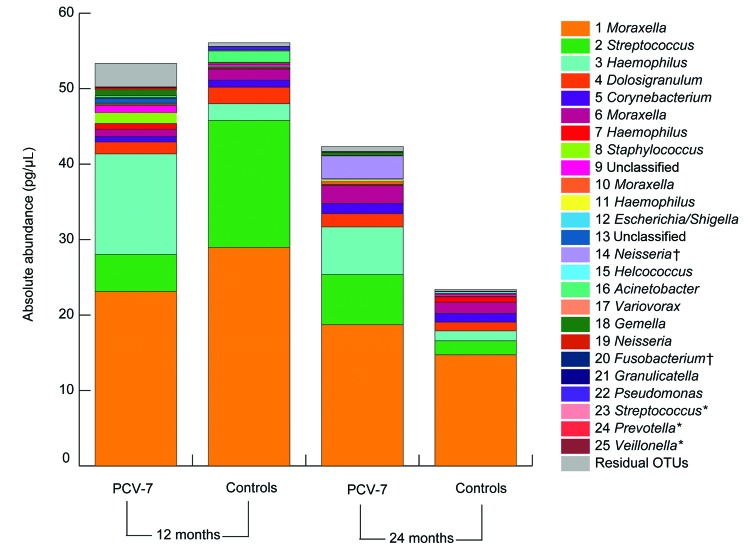
Mean absolute abundances of operational taxonomic units (OTUs) in children vaccinated with 7-valent pneumococcal conjugate vaccine and control children at 12 and 24 months of age. The 25 most abundant OTUs are represented by different colors. *OTUs that showed significantly higher abundance in vaccinated children than in controls (p<0.0003). Although not significant, an apparent higher average absolute abundance was observed for *Haemophilus* and *Staphylococcus* spp. in vaccinated children than in control children at 12 months of age. †OTUs that showed a trend toward higher abundance in vaccinated children than in controls (0.0003<p<0.05).

In univariate and multivariate linear regression models, these changes in overall community composition, variability, and bacterial diversity in vaccinated children were accompanied by significant (false-discovery rate q value <0.2, p<0.0003) increases in relative and absolute abundance of anaerobic bacteria (e.g., *Veillonella* spp., relative effect size [RES] 3.90, 95% CI 2.13–7.17; *Prevotella* spp., RES 7.24, 95% CI 4.06–12.94; unclassified *Bacteroidetes* spp., RES 2.41, 95% CI 1.28–4.54; and *Leptotrichia* spp., RES 3.31, 95% CI 1.78–6.16) as well as increases in relative and absolute abundance of several streptococcal OTUs (RES 4.53, 95% CI 2.48–8.30). A trend (0.005<p< 0.05) toward higher abundance of gram-positive *Actinomyces* spp. (RES 3.00, 95% CI 1.60–5.62) and *Rothia* spp. (RES 2.43, 95% CI 1.29–4.58), the gram-negative *Neisseria* spp. (RES 2.12, 95% CI 1.13–3.99), and the anaerobes *Fusobacterium* spp. (RES 1.93, 95% CI 1.02–3.64) and *Megasphaera* spp. (RES 1.96, 95% CI 1.03–3.71) was also observed after vaccination with PCV-7. In addition, we found apparent higher absolute abundance of *Haemophilus* (RES 1.33, 95% CI 0.73–2.44) and *Staphylococcus* (RES 1.56, 95% CI 0.83–2.93) species in vaccinated children at age 12 months (Figure 3). At 24 months of age, differences between vaccinees and controls had largely disappeared.

Although antimicrobial drug use was low, we observed a trend (0.01<p< 0.05) toward decreased relative abundance of *Dolosigranulum* (RES 0.28, 95% CI 0.061–1.33) and *Corynebacterium* (RES 0.28, 95% CI 0.061–1.30) and increased abundance of *Staphylococcus* (RES 6.29, 95% CI 1.38–28.77) in children who received antimicrobial drugs in the month before sampling.

### Microbial Inference Network in Controls and Vaccinees

**Figure 4:**
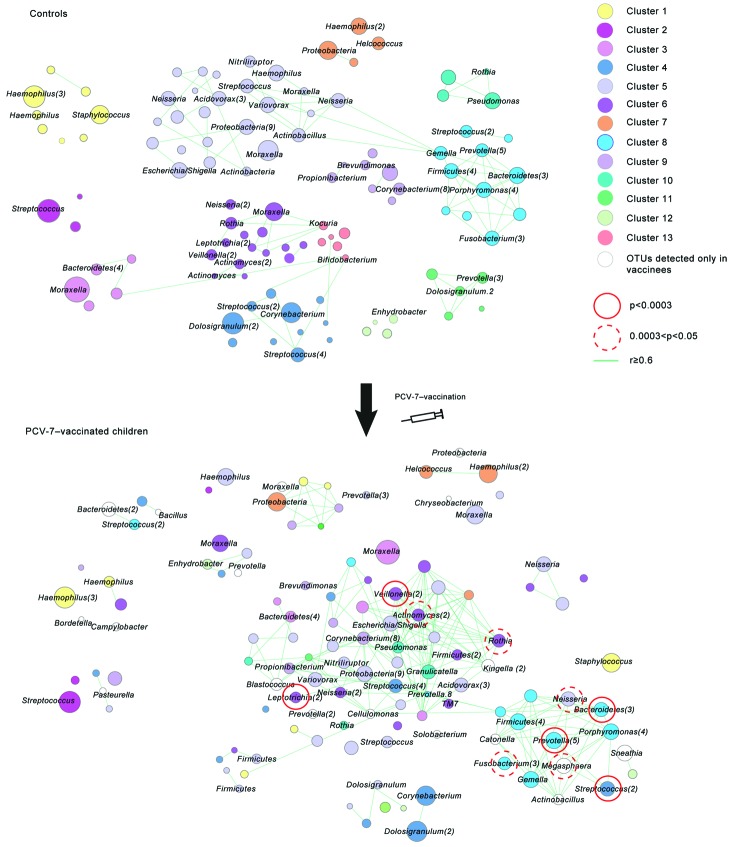
Microbial association network between operational taxonomic units (OTUs) in nonvaccinated children (controls) and children at 12 months of age who were vaccinated with 7-valent pneumococcal conjugate vaccine (PCV-7). Hierarchical clustering with average linkage and Pearson correlation distance is used to identify patterns of co-occurrence or similar abundance patterns between OTUs in the complete sample set of controls and PCV-7–vaccinated children. Results are depicted in a microbial association network. Lines connecting particular OTUs depict positive correlations (correlation coefficient r ≥0.6) between individual OTUs. Clusters of OTUs are discriminated by different colors. To enable visualization of shifts in cluster composition, OTUs in PCV-7–vaccinated children are colored according to the cluster they originated from in control children. Node sizes reflect average relative abundance of the OTU in the selected population (i.e., PCV-7–vaccinated or controls by using a log_2_ scaling. OTUs that were significantly higher in vaccinated children are indicated by red circles around nodes. For visualization purposes, we did not depict all OTU names at the nodes. Also, if multiple OTUs of the same genus clustered together, we depicted only 1 node of that genus and indicated the number of representing OTUs for that genus in parentheses. In 12 month-old children (controls), we identified 13 OTU clusters. *Haemophilus influennzae* and *Staphylococcus aureus c*lustered together in a small cluster distant from the other OTUs (cluster 1). *Streptococcus* *pneumoniae* formed, together with 2 other OTUs, a separate cluster that was also distant from the other OTUs (cluster 2). Cluster 3 contained, among others, the largest *Moraxella catarrhalis* OTU. Clusters 4–13 represent the remaining clusters and showed on average more OTUs per cluster and more interrelatedness with one another. Clear shifts in cluster composition and distribution between vaccinated and unvaccinated children were also observed. *Staphylococcus aureus* drifted from cluster 1 in controls toward cluster 8 in vaccinated children. This particular cluster increased in vaccinees because of increased abundance of OTUs already present in that cluster and because of emergence of new OTUs within the cluster. In addition, after vaccination, cluster 3 including *Moraxella catarrhalis* became part of 1 large cluster, which was composed mostly of OTUs in clusters 4–6, 9, and 10. The 10 OTUs that had expanded in vaccinated children all originated from clusters 6 and 8, or were newly emerged, such as *Megasphaera* spp.

Because microbial ecosystems form interacting networks of microorganisms, the presence (or abundance) of 1 type of bacteria will most likely affect the presence of others. To obtain better insight into the effect of vaccination on the bacterial community structure, we evaluated the effect of vaccination with PCV-7 on the microbial interaction network by using network inference analysis (Figure 4). OTUs were hierarchically clustered and displayed with their Pearson correlation by using Cytoscape V2.8.2 for control (Figure 4, panel A) and vaccinated (Figure 4, panel B) children. At the age of 12 months, children showed clear shifts in cluster distribution, composition, and interrelatedness after vaccination with PCV-7. In general, as a consequence of vaccination with PCV-7, several independent clusters observed in controls merged into 1 large cluster in vaccinees: this cluster included gram-negative anaerobes (*Prevotella*, *Veillonella*, and *Fusobacterium* spp.) as well as *Actinomyces* and *Neisseria* spp. and several streptococcal species. Bacteria that had expanded as a consequence of vaccination all belonged to the merged cluster or a single distinct cluster containing mostly *Prevotella*, unclassified *Bacteroidetes*, *Fusobacterium*, *Streptococcus*, and *Neisseria* spp. (cluster 8). The cluster containing the predominating potential pathogens *Staphylococcus* and *Haemophilus* spp. in controls (cluster 1) was divided in vaccinees because of changed behavior, in particular that of the *Staphylococcus* spp. OTU.

## Discussion

The novelty of the present study was use of deep-sequencing analyses. By using these analyses, we gained a far broader insight into the effect of PCV-7 on bacterial carriage at the ecologic niche of pneumococci without restricting selection to cultivable or well-known potential pathogens. We showed that vaccination with PCV-7 has a marked effect on the complete microbiota composition of the upper respiratory tract in children. This effect goes far beyond the shifts in pneumococcal serotypes distribution (*7*,*15*) and well-known potential pathogens reported (*8*). Vaccination with PCV-7 resulted in a shift in bacterial community composition and structure, with an increase in presence or abundance of several anaerobes, such as *Veillonella*, *Prevotella*, *Fusobacterium*, and *Leptotrichia* species; gram-positive bacteria, such as *Actinomyces* and *Rothia* species, and nonpneumococcal streptococci; and gram-negative *Neisseria* species.

Shifts in newly acquired or expanded OTUs concern mainly commensal organisms that are in general more predominantly present in the oropharynx than in the nasopharynx (*28*,*29*). Because the reduction in carriage of the 7 specific pneumococcal serotypes after PCV-7 administration correlated highly with emergence and expansion of these oropharyngeal types of species, this finding might suggest that after eradication of a common colonizer, such as vaccine serotype pneumococci, momentum is created for species from surrounding regions to colonize or expand in the vacant nasopharyngeal niche. In support of this hypothesis, Tano et al. (*30*) reported negative associations between *S. pneumoniae*, particularly PCV-7 serotypes, and other streptococcal species in healthy young children. Moreover, Laufer et al. (*31*) reported negative associations between *S. pneumoniae* and the presence of *Veillonella*, *Neisseria*, *Rothia*, and *Actinomyces* spp. in nasal swab specimens from children with upper respiratory tract symptoms, a finding that is consistent with the influx pattern of bacteria we observed after vaccination with PCV-7.

In addition to this shift in microbiota profiles, we also observed increased bacterial diversity and interindividual variability after vaccination with PCV-7. This influx or outgrowth of anaerobes and other bacteria might lead to a disequilibrium with the host. These species might be at a disadvantage again when nonvaccine serotypes fill in the gap, which would lead to a restored host-microbiome equilibrium with the host. This hypothesis could explain why we observed the strongest PCV-7 effect on microbiota in children at 12 months of age (1 month after administration of the last PCV-7 dose) and not at 24 months, because serotype replacement has already become apparent at this later time.

The mechanisms and consequences of this change in community composition and structure after vaccination with PCV-7 remain mostly speculative. In general, temporary disequilibria of bacterial composition (dysbiosis) are associated with an increased risk for disease, as has been shown for the gut (*14*) and oral niches (*32*). Moreover, the combination of some of the emerging bacteria (*Veillonella*, *Actinomyces*, *Rothia*, and *Neisseria* spp.) are associated with increased risk for otitis media (*31*). Nevertheless, in our study, we did not obtain samples during respiratory tract infections and were therefore unable to link observed changes in microbiota structure with susceptibility to respiratory tract infections. Therefore, short-term and long-term surveillance during health and disease seems warranted to understand the full implications of vaccine-induced changes in microbiota structure.

Although increased presence or abundance of *S. aureus* and *H*. *influenzae* at 12 months of age was not significant in this subset of children, we observed an increase in culture-proven *S. aureus* carriage in the original randomized controlled trial (*18*), as well as further increases in culture-proven *S. aureus* and *H. influenzae* carriage observed in surveillance studies 3–5 years after PCV-7 implementation in the Netherlands (*8*). These findings are consistent with negative associations between *S. pneumoniae* (particularly PCV-7 serotypes) and *S. aureus* (*33*,*34*) and *H. influenzae* (*35*–*37*) observed in healthy nonimmunized children. Nontypeable *H. influenzae* and *S. aureus* were also more frequently isolated from persons with acute otitis media after introduction of PCV-7 in national immunization programs (*38*–*40*), which indicates that carriage may reflect disease dynamics. Together with *S. pneumoniae* nonvaccine serotype replacement, these effects may further jeopardize the net health benefit of vaccinations with PCV.

Some limitations of our study should be recognized. First, this study was limited to a representative subset of the original study of 1,003 infants. Second, to avoid seasonal bias (*3*) in microbiota composition, we analyzed samples from only the winter season. Third, children who received antimicrobial drugs before sampling were not excluded from the analyses because only a small number of children received these drugs and we observed no correlation between antimicrobial drug use and vaccination with PCV. Furthermore, the observed associations between antimicrobial drug use and microbiota composition were also different from the vaccination effect of PCV-7.

One strength of this study was the randomized controlled study design, which enabled us to attribute changes in microbiota profiles directly to the conjugate vaccine independent of secular trends or other external confounders. Furthermore, recruitment in this study was completed well before implementation of PCV-7 in the Dutch vaccination program for newborns, and vaccine-induced changes in this randomized controlled trial setting might therefore become more apparent in the open population several years after introduction due to herd effects (*5*,*7*)

Our study indicates that vaccination against a common colonizer affects microbiota composition and structure. This finding underlines the need for more detailed understanding of microbiota dynamics and interactions between its inhabitants. Overall, because infants might be vulnerable to community disruptions and dysbiosis, we recommend that new trials, such as studies on efficacy of broader pneumococcal coverage vaccines, consider the effect of vaccination on the commensal flora in its totality instead of only on a single species.

Technical Appendix Supplemental methods.
