# Supplementary material for: Seven-Valent Pneumococcal Conjugate Vaccine and Nasopharyngeal Microbiota in Healthy Children
Source: Emerg Infect Dis. 2014 Feb;20(2):201–10. doi: 10.3201/eid2002.131220 (PMC3901477; doi:10.3201/eid2002.131220)
Supplement: Technical Appendix — Supplemental methods. [file 13-1220-Techapp-s1.pdf]

Article DOI: http://dx.doi.org/10.3201/eid2002.131220

Seven-valent Pneumococcal Conjugate Vaccine and Nasopharyngeal Microbiota in Healthy Children

Supplemental Methods

Sample Collection and Storage

We performed a randomized controlled trial of 1,003 children to study the efficacy of reduced-dose schedules of seven-valent pneumococcal conjugate vaccine (PCV-7) on nasopharyngeal pneumococcal carriage as described (*1*). Transnasally obtained nasopharyngeal swabs and questionnaires were collected at 6 weeks, 6, 12, 18, and 24 months of age. For the current study, we selected nasopharyngeal samples of children who had received PCV-7 vaccination at 2, 4, and 11 months of age, and PCV-7–unvaccinated children (controls) who did not receive 1 dose of PCV-7 until after the end of the trial. To avoid seasonal bias, we selected only those children sampled at 12 and 24 months of age during October 2006–January 2006–2007 and September–January 2007–2008, respectively (*2*). To avoid interference from background DNA, only those children with sufficient bacterial density (i.e., samples with DNA levels ≥1 pg/µL) were selected for 454 pyrosequencing (*3*). This resulted in inclusion of 97 PCV-7–vaccinated children and 103 control children sampled at 12 and 24 months of age.

Samples were collected during home visits and detailed metadata were obtained by questionnaires, including the presence of siblings, day-care attendance and use of antimicrobial drugs before sampling. All children were considered healthy and nonfebrile during home visits.

Nasopharyngeal swabs were obtained by using a transnasal approach with a flexible, sterile, dry cotton-wool swab (TranswabPernasal Plain; Medical Wire and Equipment Co., Ltd., Corsham, UK) and immediately inoculated into transswab modified Amies medium, 483CE (Copan Diagnostics Inc., Murrieta, CA, USA), transported to the laboratory, and stored within 24 hours at –80°C until further analyses.

Ethics Statement

This randomized controlled trial was approved by an acknowledged Dutch National Ethics Committee (Stichting Therapeutische Evaluatie Geneesmiddelen; http://www.stegmetc.org), and the trial was undertaken in accordance with European Statements for Good Clinical Practice, which included the provisions of the Declaration of Helsinki of 1989. Parents living in the western part of the Netherlands were provided with written information about the study and asked to participate. Before enrollment, written informed consent was obtained from both parents of each participant.

Detection of Bacteria by Using Conventional Culture Approach

Nasopharyngeal swabs were plated onto a 5% sheep blood agar plate containing 5 mg/L gentamicin, a chocolate agar plate, and a *Haemophilus* chocolate agar plate. Plates were incubated at 35°C for 48 h in aerobic conditions. Blood agar plates containing 5 mg/L gentamicin and chocolate agar plates were incubated in an atmosphere of increased CO_2. Cultures were checked daily for the growth of *Streptococcus pneumonia*, *Haemophilus influenza*, *Moraxella catarrhalis*, and *Staphylococcus aureus*, and species were confirmed by using conventional methods (*4*). Pneumococcal serotyping was performed by using the capsular swelling method (Quellung reaction) with type-specific antisera from the Statens Seruminstitut (Copenhagen, Denmark).

Construction of Phylogenetic Library

The subset was processed for 454 GS-FLX-Titanium sequencing of the 16S small subunit rDNA gene. The 16S rDNA gene is a conserved gene with variable regions among bacteria.

Extraction of Bacterial DNA

For each sample, 0.3 g zirconium beads (diameter 0.1 mm, catalog no. 11079101z; Biospec Products, Bartlesville, OK, USA), 200 μL phenol (Phenol solution BioUltra; TE-saturated; Sigma-Aldrich, St. Louis, MO, USA), and 150 μL lysis buffer (Mag Mini DNA Isolation Kit; AGOWA, Berlin, Germany) were added to 200 μL of sample. All compounds were mixed and cells were mechanically lysed by using a Mini-beadbeater (Mini-beadbeater 16; Biospec Products). DNA was purified by using magnetic beads as part of the Mag Mini DNA Isolation Kit (AGOWA). DNA was washed and eluted in 50 μL elution buffer as recommended by the manufacturer.

Real-time PCR for Bacterial DNA

Quantity of bacterial DNA was measured for each sample by using quantitative PCR and a universal primer-probe set specific for the 16S rDNA gene as described (*2*) containing forward primer 16S-F1 (5′-CGA AAG CGT GGG GAG CAA A –3′), reverse primer 16S-R1 (5′-GTT CGT ACT CCC CAG GCG G-3′) and probe 16S-P1 (FAM-ATT AGA TAC CCT GGT AGT CCA-MGB). The PCR mixture consisted of 15 μL of 2× master mixture (Universal Mastermix, catalog no. GMO-UN-A100; Europe Diagenode SA, Liège, Belgium), 1 μL of each primer (10 μmol/L), 1 μL of probe (5 μmol/L), 9.5 μL DNAase-free water, and 2.5 μL of template DNA. The machine used for the amplifications was a 7500 Fast Real-Time PCR System (catalog no. 4351107, Applied Biosystems, Foster City, CA, USA) with the following conditions: 2 min at 50°C and 10 min at 95°C, followed by 45 cycles of 15 s at 95°C and 1 min at 60°C. Obtained cycle threshold values were related to a standard curve of bacterial DNA obtained from human saliva spiked with DNA of 6 bacterial species (*Streptococcus mutans*, *Fusobacterium nucleatum*, *Porphyromonas gingivalis*, *Porphyromonas catoniae*, *Propionibacterium propionicum*, and *Tannerella forsythia*). We used a standard DNA range of 0.1 pg/μL through 1 ng/μL. Only samples with DNA levels ≥1 pg/μL were selected for 454 pyrosequencing to avoid interference of background DNA (*3*).

Preparation of Amplicon Library

An amplicon library was generated by amplification of the V5–V7 hypervariable region of this gene as described (*2*,*3*). We used forward primer 785F (5′-GGA TTA GAT ACC CBR GTA GTC-3′) and reverse primer 1061R (5′-TCA CGR CAC GAG CTG ACG AC-3′). The primers were fitted with the 454 Life Sciences Adaptor A (forward primer) and B (reverse primer) and fused to the 5′ end of the 16S rDNA bacterial primer sequences. The reverse primer also included a unique decanucleotide sample identification key. The amplification mixture contained 2 units of Pfu Ultra II Fusion HS DNA polymerase (Stratagene, La Jolla, CA, USA) and 1× *PfuUltra* II Reaction Buffer (Stratagene), 200 μmol/L dNTP PurePeak DNA Polymerase Mix (catalog no. NU606001; Pierce Nucleic Acid Technologies Milwaukee, WI, USA), and 0.2 μmol/L of each primer. After denaturation (94°C for 2 min), 30 cycles were performed that consisted of denaturation (94°C for 30 s), annealing (50°C for 40 sec), and extension (72°C for 80 sec). In addition, samples with DNA levels ≤pg/μL of DNA were cycled 35 times instead of 30 times by using the same protocol.

Amplicons were size checked and quantified by gel electrophoresis and Quant-iT PicoGreen dsDNA Assay (Invitrogen, Carlsbad, CA, USA) by using a Tecan Infinite M200 (Tecan Group Ltd., Männedorf, Switzerland). Amplicons of the individual samples were equimolar pooled, purified by agarose gel electrophoresis, and isolated from gels by using the QIAquick Gel Extraction Kit Protocol (QIAGEN, Hilden, Germany). The amplicon library was sequenced unidirectionally by using the UCL Genomics London, GS-titantium-sequencer 454 Life Sciences (Roche, Branford, CT, USA) and generated ≈1.5 million sequences. Mock communities were included in each amplicon library for technical control over quality of sequence data.

Data Processing

The obtained sequences were processed and classified by suing modules implemented in Mothur version 1.20.0 software platform according to described methods (*5*–*9*). FASTA-formatted sequences and corresponding quality scores were extracted from .sff data file generated by the GS-FLX-Titanium sequencer by using the GS Amplicon Software Package (Roche, Branford, CT). Sequences were de-noised by using a pseudo-single linkage algorithm with the goal of removing sequences that might have been caused by pyrosequencing errors (pre.cluster command) (*10*). Raw sequences were trimmed and checked for quality and those containing ambiguous base call in the sequence, >1 error in the primer or any sequence error in the barcode, more than 8 polyhomomers, or a q-window <35 bp and a length >500 bp or <200 bp were deleted from the dataset. Chimeric sequences were detected and removed by using the chimera.slayer command (*11*). High-quality aligned sequences (1.016.934 sequences, mean ± SD 2,561 ± 767 sequences/sample) were classified by using the RDP-II naive Bayesian Classifier (*9*).

Aligned sequences were clustered into operational taxonomic units (OTUs, defined by 97% similarity) by using the average linkage clustering method. An OTU is the smallest accurate taxonomic classification level obtained by using 16S rDNA gene sequencing and enables differentiation just beyond genus level. For each of the samples, rarefaction curves were plotted and community diversity parameters (Shannon diversity index, Chao1, and Simpson’s) were calculated. Sequence data were subsequent subjected to weighted UniFrac analysis by using the UniFrac module implemented in Mothur (*5*). The UniFrac metric is a proxy for the distance between different microbial communities and takes into account the phylogenetic relatedness of lineages in each sample. The phylogenetic dendrogram for the UniFrac analyses was obtained by using FastTree (*12*). For all samples, we calculated the

presence and relative and absolute abundance of all OTUs. Relative abundance was calculated as the proportion of sequences assigned to a specific OTU divided by the overall number of obtained sequences per sample. In addition, to take the absolute abundance into account, we multiplied the relative abundance of an OTU by the obtained bacterial load per sample measured by quantitative PCR.

Statistical Analyses

Data analyses were performed by using R version 2.7 (http://cran.r-project.org/bin/windows/base/old/2.7.1/), Excel 2011 (Microsoft, Redmond, WA, USA), and SPSS version 15.0 (SPSS Inc., Armonk, NY, USA). In all statistical analyses, we used the normalized and logarithmically (log10 scale) transformed relative and absolute abundance OTU matrix. We used Pearson χ^2 tests to test for comparability of baseline characteristics between PCV-7–vaccinated children and control children (SPSS Statistics version 15.0). To visualize the weighted UniFrac dendrogram in relation to metadata, we used online iTOL version 2 software (*13*). We also used univariate and multivariate linear regression models (function Im and analysis of variance in software package R 2.7), to study the effect of PCV-7 vaccination on microbiota profiles. We adjusted for known risk factors for bacterial colonization (i.e., daycare attendance, antimicrobial drug use within 1 month before sampling, and presence of siblings.

Because of the high number of comparisons, associations were considered statistically significant after correction for multiple testing by determining the false-discovery rate (q value 0.2). To obtain standardized effect sizes and their 95% confidence intervals, we performed a standardizing calculation of the regression coefficient by using the formula $\beta = b \times 1/sy$, where b is the regression coefficient and sy is the standard error of y. Because PCV-7 vaccination is a dichotomous variable (PCV-7 vaccination yes/no), we only corrected for the SE of y and not for the SE of x. The relative effect sizes can be considered as relative ratios whereby 1 indicates no effect, >1 indicates higher abundance or present, and <1 indicates less abundance or present in vaccinated children compared with controls. Interindividual variability was calculated by using Pearson correlations between vaccinated and control children at 12 months and 24 months of age. Correlation matrices of the groups were compared afterwards: means and SDs were calculated. Significance of the differences was calculated by using t-tests.

We used nonmetric multidimensional scaling to compare microbiota profiles for dissimilarities, and Euclidean distances were used to locate each sample in a low-dimensional

space. The relative abundance OTU matrix was used as input for nonmetric multidimensional scaling analyses. OTUs were then clustered hierarchically by using average linkage and Pearson correlation and absolute Pearson correlation distance on the relative abundance OTU matrix. The optimal number of clusters was identified by using the Silhouette index. Clusters were calculated and located by using the plug-in Clustermaker in Cytoscape version 2.8.2. Multivariate analyses, OTU clusters, and Pearson correlations between OTUs were displayed by using Cytoscape version 2.8.2 (*14*) to obtain a network model.

References

<jrn>1. van Gils EJ, Veenhoven RH, Hak E, Rodenburg GD, Bogaert D, Ijzerman EP, et al. Effect of reduced-dose schedules with 7-valent pneumococcal conjugate vaccine on nasopharyngeal pneumococcal carriage in children: a randomized controlled trial. JAMA. 2009;302:159–67. PubMed http://dx.doi.org/10.1001/jama.2009.975</jrn>

<jrn>2. Bogaert D, Keijser B, Huse S, Rossen J, Veenhoven R, van Gils E, et al. Variability and diversity of nasopharyngeal microbiota in children: a metagenomic analysis. PLoS ONE. 2011;6:e17035. PubMed http://dx.doi.org/10.1371/journal.pone.0017035</jrn>

<jrn>3. Biesbroek G, Sanders EA, Roeselers G, Wang X, Caspers MP, Trzciński K, et al. Deep sequencing analyses of low density microbial communities: working at the boundary of accurate microbiota detection. PLoS ONE. 2012;7:e32942. PubMed http://dx.doi.org/10.1371/journal.pone.0032942</jrn>

<bok>4. Versalovic J, Carroll KC. Manual of clinical microbiology. Washington (DC): American Society for Microbiology Press; 2011.</bok>

<jrn>5. Lozupone C, Knight R. UniFrac: a new phylogenetic method for comparing microbial communities. Appl Environ Microbiol. 2005;71:8228–35. PubMed http://dx.doi.org/10.1128/AEM.71.12.8228-8235.2005</jrn>

<jrn>6. Schloss PD, Westcott SL, Ryabin T, Hall JR, Hartmann M, Hollister EB, et al. Introducing mothur: open-source, platform-independent, community-supported software for describing and comparing microbial communities. Appl Environ Microbiol. 2009;75:7537–41. PubMed http://dx.doi.org/10.1128/AEM.01541-09</jrn>

<jrn>7. Shendure J, Ji H. Next-generation DNA sequencing. Nat Biotechnol. 2008;26:1135–45. PubMed http://dx.doi.org/10.1038/nbt1486</jrn>

<jrn>8. Whitman WB, Coleman DC, Wiebe WJ. Prokaryotes: the unseen majority. Proc Natl Acad Sci U S A. 1998;95:6578–83. PubMed http://dx.doi.org/10.1073/pnas.95.12.6578</jrn>

<jrn>9. Cole JR. The Ribosomal Database Project (RDP-II): previewing a new autoaligner that allows regular updates and the new prokaryotic taxonomy. Nucleic Acids Res. 2003;31:442–3. PubMed http://dx.doi.org/10.1093/nar/gkg039</jrn>

<jrn>10. Huse SM, Welch DM, Morrison HG, Sogin ML. Ironing out the wrinkles in the rare biosphere through improved OTU clustering. Environ Microbiol. 2010;12:1889–98. PubMed http://dx.doi.org/10.1111/j.1462-2920.2010.02193.x</jrn>

<jrn>11. Haas BJ, Gevers D, Earl AM, Feldgarden M, Ward DV, Giannoukos G, et al. Chimeric 16S rRNA sequence formation and detection in Sanger and 454-pyrosequenced PCR amplicons. Genome Res. 2011;21:494–504. PubMed http://dx.doi.org/10.1101/gr.112730.110</jrn>

<jrn>12. Price MN, Dehal PS, Arkin AP. FastTree 2–approximately maximum-likelihood trees for large alignments. PLoS ONE. 2010;5:e9490. PubMed http://dx.doi.org/10.1371/journal.pone.0009490</jrn>

<jrn>13. Letunic I, Bork P. Interactive Tree Of Life (iTOL): an online tool for phylogenetic tree display and annotation. Bioinformatics. 2007;23:127–8. PubMed http://dx.doi.org/10.1093/bioinformatics/btl529</jrn>

<jrn>14. Cline MS, Smoot M, Cerami E, Kuchinsky A, Landys N, Workman C, et al. Integration of biological networks and gene expression data using Cytoscape. Nat Protoc. 2007;2:2366–82. PubMed http://dx.doi.org/10.1038/nprot.2007.324</jrn>

Technical Appendix Table 1. Top 25 operational taxonomic units found in the nasopharyngeal samples of 200 children sampled at 12 and 24 months of age after vaccination with 7-valent pneumococcal conjugate vaccine*

Phylum	Genus	No. present (% of infants)†	12-mo relative abundance‡	Absolute abundance (pg/μL)§	No. present (% of infants)†	24-mo relative abundance‡	Absolute abundance (pg/μL)§
Proteobacteria	*Moraxella*	194 (97)	58.115	26.56	197 (98.5)	58.704	16.64
Firmicutes	*Streptococcus*	188 (94)	14.003	11.52	170 (85)	10.574	4.57
Proteobacteria	*Haemophilus*	110 (55)	15.726	12.44	108 (54)	14.669	6.27
Firmicutes	*Dolosigranulum*	158 (79)	7.499	2.29	169 (84.5)	8.556	1.67
Actinobacteria	*Corynebacterium*	133 (66.5)	4.613	1.17	148 (74)	5.611	1.63
Proteobacteria	*Moraxella*	42 (21)	11.884	4.59	57 (28.5)	12.911	5.64
Proteobacteria	*Haemophilus*	34 (17)	5.256	2.02	49 (24.5)	8.793	1.55
Firmicutes	*Staphylococcus*	49 (24.5)	6.621	2.45	39 (19.5)	4.298	0.09
Proteobacteria	Unclassified	17 (8.5)	17.525	6.05	11 (5.5)	8.791	2.41
Proteobacteria	*Moraxella*	59 (29.5)	2.481	0.46	73 (36.5)	1.798	0.65
Proteobacteria	*Haemophilus*	14 (7)	6.517	1.28	17 (8.5)	5.569	1.92
Proteobacteria	*Escherichia/Shigella*	100 (50)	0.444	0.04	122 (61)	0.449	0.02
Proteobacteria	Unclassified	77 (38.5)	0.533	0.04	109 (54.5)	0.410	0.01
Proteobacteria	*Neisseria*	14 (7)	2.138	0.46	4 (2)	10.194	31.64
Firmicutes	*Helcococcus*	17 (8.5)	0.617	0.33	34 (17)	1.602	0.78
Proteobacteria	*Acinetobacter*	26 (13)	1.139	5.74	30 (15)	1.142	0.02
Proteobacteria	*Variovorax*	38 (19)	0.858	0.02	39 (19.5)	0.548	0.02
Firmicutes	*Gemella*	46 (23)	0.685	1.15	36 (18)	0.597	0.82
Proteobacteria	*Neisseria*	59 (29.5)	0.427	0.36	45 (22.5)	0.580	0.09
Fusobacteria	*Fusobacterium*	20 (10)	0.350	0.27	12 (6)	3.132	0.53
Firmicutes	*Granulicatella*	58 (29)	0.385	0.17	33 (16.5)	0.354	0.04
Proteobacteria	*Pseudomonas*	20 (10)	1.577	1.79	11 (5.5)	0.100	0.00
Firmicutes	*Streptococcus*	46 (23)	0.299	0.07	55 (27.5)	0.210	0.04
Bacteroidetes	*Prevotella*	46 (23)	0.157	0.02	25 (12.5)	0.094	0.03
Firmicutes	*Veillonella*	44 (22)	0.138	0.02	25 (12.5)	0.508	0.05
Bacteroidetes	Unclassified	22 (11)	1.295	0.12	13 (6.5)	0.279	0.03
Proteobacteria	*Curvibacter*	50 (25)	0.244	0.01	62 (31)	0.198	0.01
Proteobacteria	*Acidovorax*	43 (21.5)	0.364	0.01	46 (23)	0.276	0.01
Firmicutes	Unclassified	15 (7.5)	1.422	3.76	7 (3.5)	0.138	0.05
Firmicutes	*Streptococcus*	7 (3.5)	0.431	0.03	15 (7.5)	1.260	0.06
Fusobacteria	*Sneathia*	2 (1)	0.175	18.52	1 (0.5)	0.001	0.02
Proteobacteria	*Moraxella*	4 (2)	0.035	8.88	3 (1.5)	0.024	0.66
Bacteroidetes	*Porphyromonas*	17 (8.5)	0.006	1.68	13 (6.5)	0.007	0.07
Bacteroidetes	*Porphyromonas*	16 (8)	0.009	0.93	10 (5)	0.004	0.01
Bacteroidetes	Unclassified	27 (13.5)	0.003	0.42	20 (10)	0.003	0.07
Proteobacteria	*Actinobacillus*	11 (5.5)	0.010	0.24	14 (7)	0.003	0.05
Bacteroidetes	*Porphyromonas*	32 (16)	0.003	0.19	27 (13.5)	0.002	0.03
Cyanobacteria	*Streptophyta*	11 (5.5)	0.008	0.11	16 (8)	0.003	0.02
Firmicutes	Unclassified	12 (6)	0.011	2.60	5 (2.5)	0.002	0.03
Firmicutes	*Streptococcus*	25 (12.5)	0.004	0.17	18 (9)	0.001	0.02
Proteobacteria	*Haemophilus*	18 (9)	0.001	0.08	12 (6)	0.009	1.11
Firmicutes	Unclassified	42 (21)	0.002	0.02	48 (24)	0.001	0.00
Actinobacteria	*Actinomyces*	22 (11)	0.002	0.06	9 (4.5)	0.009	0.05
Bacteroidetes	Unclassified	1 (0.5)	0.117	0.84	1 (0.5)	0.001	0.02
Fusobacteria	*Streptobacillus*	14 (7)	0.005	0.67	8 (4)	0.005	0.10
Firmicutes	*Parvimonas*	2 (1)	0.001	0.01	6 (3)	0.017	0.21
Actinobacteria	*Rothia*	39 (19.5)	0.002	0.03	20 (10)	0.001	0.01
Proteobacteria	*Pelomonas*	29 (14.5)	0.001	0.01	33 (16.5)	0.001	0.01
Actinobacteria	*Propionibacterium*	22 (11)	0.001	0.01	49 (24.5)	0.001	0.00
Actinobacteria	*Nitriliruptor*	38 (19)	0.001	0.01	38 (19)	0.001	0.00

*Shown are the top 25 operational taxonomic units (OTUs) observed in the population with respect to presence (no. children), relative abundance when present (total sequences/no. persons positive for this specific OTU), and absolute abundance when present (taking into account bacterial density observed) at 12 and 24 months of age. All single sequences in samples were removed from the analyses (calculated for the average no. of sequences obtained for the samples). OTUs were significantly correlated with the corresponding culture for *M. catarrhalis*, *S. pneumoniae*, *H. influenza,* and *S. aureus*.

†No. samples in which this OTU is present (absence versus presence).

‡Average relative abundance when this OTU is present in the sample.

§Average absolute abundance when this OTU is present in the sample.

Technical Appendix Table 2. Correlations of culture results to the first operational taxonomic units of4 bacterial species in children sampled at 12 and 24 months of age after vaccination with 7-valent pneumococcal conjugate vaccine*

	p value			
OUT	*Moraxella catharralis*	*Haemophilus influenzae*	*Streptococcus pneumoniae*	*Staphylococcus aureus*
M. catharralis	0.003	0.096	0.021	0.1170
H. influenza	0.410	0.000	0.080	0.2130
S. pneumoniae	0.006	0.953	0.000	0.3270
S. aureus	0.05	0.151	0.222	0.0001

*Operational taxonomic units (OTUs) are based on 97% similarity in nucleotide composition and therefore enable differentiation just beyond genus level. OTUs cannot discriminate between species or strains, although several OTUs may exist for a specific genus. To illustrate, for the genus *Moraxella* we observed several OTUs (online Technical Appendix Table 2). To be able to discriminate between *M. catarrhalis* and other *Moraxella* species, we correlated culture results to the presence of *Moraxella* OTUs. We also correlated *S. pneumoniae*, *H. influenzae*, and *S. aureus* culture results to the subsequent OTUs. Results are shown in this table. We observed a strong correlation between the culture results and the first OTUs of *Moraxella*, *Streptococcus*,*Haemophilus*, and *Staphylococcus* species, respectively (p<0.001), indicating a strong representation of the pathobionts in these OTUs. This analyses was performed with a multivariate regression model, corrected for multiple testing (false-discovery rate), antimicrobial drugs (orally or intravenously administered) within 1 mo before sampling, and crowding factors (i.e., daycare attendance, defined as at least 4 h/wk with more >1child from a different family and the presence of siblings).

Technical Appendix Table 3. Microbiota characteristics of children vaccinated with 7-valent pneumococcal conjugate vaccine and unvaccinated children (controls) at 12 and 24 months of age*

	12 months			24 months		
Characteristic	PCV-7, n = 97	Control, n = 103	p value	PCV-7 n = 97	Control, n = 103	p value
Bacterial density, pg/µL (95% CI)†	52.8 (30.6–74.9)	56·1 (30.5–81.7)	NS	42.8 (19.7–65.9)	23.4 (15.8–31.0)	NS
Relative abundance of gram-negative bacteria, % (95% CI)	76.0 (70.9–81.2)	73.3 (68.1–78.6)	NS	75.3(70.4 – 80.3)	78·4 (74.2–82.6)	NS
Absolute abundance of gram-negative bacteria, pg/µL (95% CI)	40.8 (21.7–59.8)	35.3 (20.7–50.0)	NS	31.6 (14.2–49.1)	18·8(12.3–25.3)	NS
Mean (SD) no. OTUs per sample	24 (12)	19 (9)	0.001	23 (13)	21 (9.4)	NS
Shannon diversity index (SD)	0.96 (0.57)	0.81 (0.47)	NS	0.94 (0.5)	0.30 (0.47)	NS
Interindividual variability‡	0.43	0.47	0.0001	0.45	0.47	0.02

*PC-7, 7-valent pneumococcal conjugate vaccine; NS, not significant (p>0.1); OTUs, operational taxonomic units. p values were determined by using the χ^2 test. p values are shown when there was a trend (p = 0.1–0.05) or a significant difference (p<0.05).
†Determined by using quantitative PCR analyses of the total bacterial load.
‡Determined by using Pearson correlations between profiles of all children within the PCV-7–vaccinated group and within the control group. Significance of correlation matrices between groups was calculated by using the Mann-Whitney test.
